# miR-6742-5p regulates the invasion and migration of lung adenocarcinoma cells via mediating FGF8/ERK12/MMP9/MMP2 signaling pathway

**DOI:** 10.18632/aging.204277

**Published:** 2023-01-10

**Authors:** Minglei Song, Xiaoying Xing

**Affiliations:** 1 Department of Thoracic Surgery, The Fourth Hospital of Hebei Medical University, Shijiazhuang 050000, Hebei, China; 2 Department of General Practice, The Second Hospital of Hebei Medical University, Shijiazhuang 050000, Hebei, China

**Keywords:** LUAD, miR-6742-5p, FGF8, ERK1/2/MMPs, tumor metastasis

## Abstract

Background: microRNAs (miRNAs) are involved in the progression of Lung adenocarcinoma (LUAD), however, the functions of miR-6742-5p in LUAD remains unknown, thereby this study was carried on.

Methods: The mRNA and miRNA expression data from the LUAD and normal control were obtained from Gene Expression Omnibus (GEO) database, TargetScan and mirDIP were applied to predict the relationship between miR-6742-5p and FGF8.Q-PCR, western blot, dual-luciferase, wound Healing and transwell assays were performed to test the functions of miR-6742-5p in LUAD.

Results: Bioinformatics analysis and dual-luciferase identified FGF8 is the target-gene of miR-6742-5p, which is declined in LUAD of human tissues and cell lines, and miR-6742-5P OE suppressed the progression of LUAD in nude mice. MiR-6742-5p OE and KD suppressed or increased the abilities of LUAD’ metastasis tested by wound healing and transwell assays H522 and PC-9 cells, these effects about miR-6742-5p OE were reversed by FGF8; miR-6742-5p OE, KD inhibited and increased the expression of FGF8 as its downstream p-ERK1/2, MMP-2/-9, these results were corrected by ERK1/2 inhibitor: Ro 67-7476; the miR-6742-5p KD increased the migrated and invaded cells and suppressed by MMPs inhibitor: S3304. These results identified the negative correlation of miR-6742-5p with FGF8-ERK1/2 signal pathway in LUAD progression.

Conclusions: We conclude that miR-6742-5p might be a regulator of LUAD progression by targeting FGF8/ERK1/2/MMPs signaling pathway, which provides a novel therapeutic target for LUAD.

## INTRODUCTION

Lung cancer is the most common malignancy with the highest morbidity and mortality rates worldwide [1]. Lung adenocarcinoma (LUAD) is one of the main subtypes of non-small cell lung cancer, accounting for 40% of all lung cancers [2]. Studies have revealed that LUAD patients exhibit a relatively high mortality rate, and the risk of distant metastasis exceeds that of local recurrence at each stage of the disease, confirming the systemic involvement of the disease [3]. Despite the recent advances in molecular diagnosis and treatment technology, the prognosis of LUAD remains not optimistic, and the risk of metastasis and recurrence remains high. Therefore, it is essential to uncover the underlying mechanism of LUAD and to find novel therapeutic targets for LUAD.

Micro ribonucleic acids (miRNAs) are a class of small non-coding RNAs that are considered to be key regulators of biological processes [4]. Abnormal expression of miRNAs has been reported to be associated with the occurrence and progression of a variety of tumors, and miRNAs can be used as biomarkers for tumor diagnosis and prognosis [5, 6]. As reported, many miRNA molecules are involved in various biological processes in the occurrence and development of multiple cancers, including cell proliferation, apoptosis, angiogenesis, migration, and invasion. For instance, the overexpression (OE) of miR-3174 significantly promotes cell proliferation and inhibits cell apoptosis by suppressing forkhead box protein O1 (FOXO1) expression in hepatocellular carcinoma [7]. MiR-192 and miR-215 target adenomatous polyposis coli and function as oncogenic miRNAs via activating the Wnt signaling pathway in gastric cancer, suggesting that miR-192 and miR-215 are potential therapeutic targets [8]. In addition, the up-regulation of miR-183-5p can promote the occurrence of colorectal cancer by regulating FOXO1, proving its potential role as biomarker for the treatment of colorectal cancer [9]. Moreover, miR-335-5p modulates cell cycle and metastasis of LUAD through targeting CCNB2 [10]. All the above reports are suggestive of the important roles of miRNAs in cancer progression. However, the function and mechanism of miR-6742-5p in LUAD are still ambiguous. The fibroblast growth factor 8 (FGF8)/FGF receptor 1 (FGFR1)/extracellular signal-regulated kinase 1/2 (ERK1/2) axis functions in the tumorigenesis in various tumors, including lung cancer, and its role involves involved in the invasion, metastasis and progression of non-neoplastic lesions. FGF8 activates FGFR1 by directly binding to it, which aggravates lung cancer and results in the phosphorylation of ERK1/2 [11–19]. Moreover, matrix metalloproteinases (MMPs) are key executors of tumor invasion, which are the downstream products of ERK1/2 signals. They inhibit tumor metastasis by degrading the fibrous or stromal tissue surrounding the tumor, thus limiting the cancer cell migration throughout the body [20–23]. Hence, FGFs, ERK1/2 and MMPs are key checkpoints for cancer suppression.

In the present study, the potential molecular mechanism of miR-6742-5p in LUAD was elucidated, which is expected to provide a novel therapeutic target for LUAD.

## RESULTS

### DEGs and DEMs in LUAD

A total of 266 DEGs between LUAD and normal tissues were retrieved from GSE140797 dataset (Supplementary Figure 1) and 257 from GSE116959 dataset (Supplementary Figure 2). In addition, 61 DEMs were retrieved from the GSE94536 dataset and subjected to hierarchical clustering analysis (Supplementary Figure 3).

**Figure 1:**
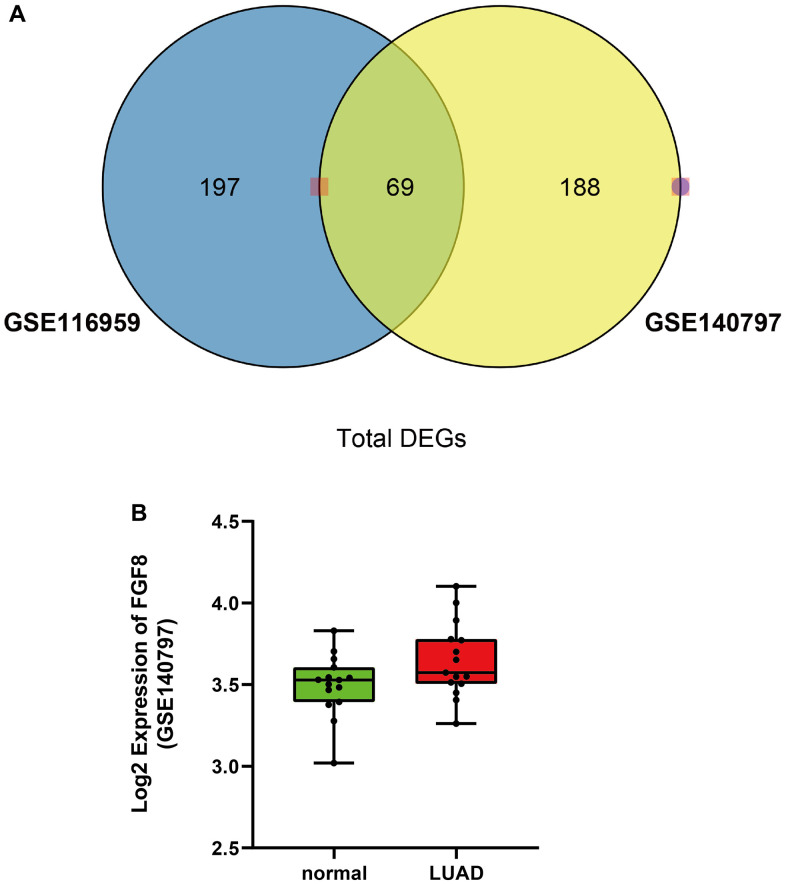
**Differentially expressed genes in both GSE116959 and GSE140797 datasets.** (**A**) A total of 69 differentially expressed genes were obtained in both GSE116959 and GSE140797 datasets. (**B**) The expression level of FGF8 in GSE140797 dataset.

**Figure 2:**
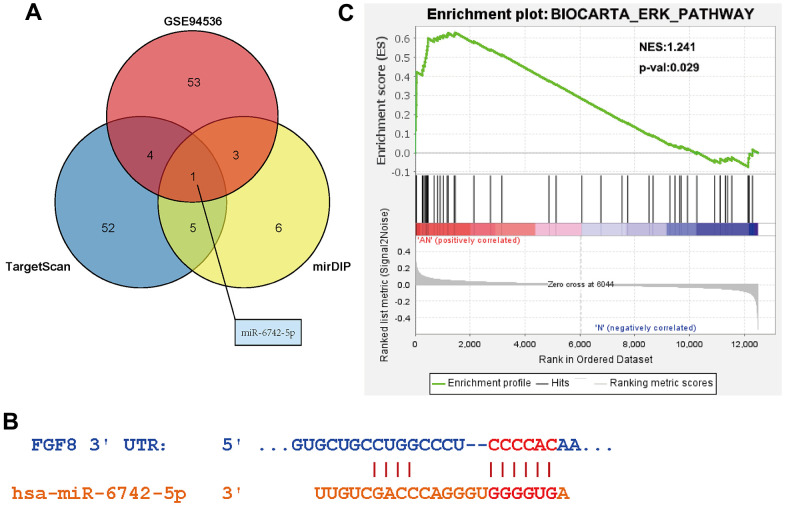
**Determine of the targeting relationship between miR-6742-5p and FGF8.** (**A**) Identification of FGF8 targeting miRNAs. (**B**) Predicted binding sites between miR-6742-5p and FGF8. (**C**) High expression of FGF8 was associated with ERK pathway.

There were 69 DEGs common to the GSE140797 and GSE116959 datasets (Figure 1A), which were thus used for subsequent analysis. Among the 69 DEGs, FGF8 gene attracted our attention, and its expression and role in LUAD were explored. The results manifested that the FGF8 expression was up-regulated in LUAD tissues (Figure 1B). Subsequently, the upstream miRNAs of FGF8 were assessed using miDIP and TargetScan databases, and the key regulatory factor of FGF8, miR-6742-5p, was filtered from the DEMs common to the three datasets, which exhibited a down-regulated expression LUAD tissues (Figure 2A). Furthermore, the binding sites between miR-6742-5p and FGF8 were obtained from target predictions on the miRDIP database (Figure 2B), and moreover, the original data were shown in Supplementary Figure 5. Ultimately, GSEA results uncovered that FGF8 gene was significantly enriched in the ERK pathway, suggesting that the high expression of FGF8 is associated with the ERK pathway (Figure 2C).

### Relative expression and role of miR-6742-5p in human LUAD and model nude mouse tissues

**Figure 3:**
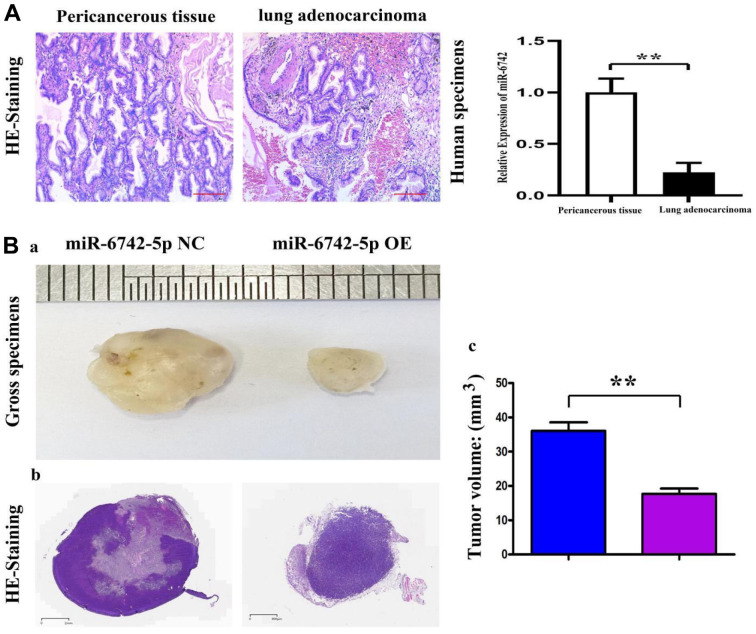
**The expression of miR-6742-5p in human lung adenocarcinoma and its roles in nude mice.** (**A**) Relative expression of miR-6742-5p in human lung adenocarcinoma and their paracancerous tissues, tested by quantitative real-time PCR. (**B**, **a**) the volume of H522 tumor in miR-6742-5p NC and OE group; (**B**, **b**) HE staining of nude mice’ tumors; (**B**, **c**) statistic data for tumor volume. (**A**) ** P=0.0001 miR-6742-5p OE group vs NC group; (**B**) **P=0.0001 miR-6742-5p OE group vs NC group.

The relative expression of miR-6742-5p in paracancerous tissues of LUAD was examined using qRT-PCR, and it was found that the miR-6742-5p expression was significantly decreased in human LUAD tissues compared with that in the corresponding paracancerous tissues (Figure 3A). Moreover, to further evaluate the roles of miR-6742-5p in LUAD, a nude mouse model of LUAD was established. BALB/c mice in the two groups were subcutaneously injected with 100 μL of cell suspension containing 1×10^7^ H522 cells per mL in sterile PBS. 4 weeks later, the tumors were collected, and it was uncovered that the tumor growth in miR-6742-5p mimic/OE group was lower than that in miR-6742-5p NC group (Figure 3Ba). As shown in Figure 3Bb, the histopathology of the nude mice examined by H&E staining demonstrated that the size and the area of tumors were obviously inhibited in miR-6742-5p mimic/OE group compared with those in miR-6742-5p NC group, data were shown in Figure 3Bc.

### Relative expression of miR-6742-5p and its role in H522 and PC-9 cells

**Figure 4:**
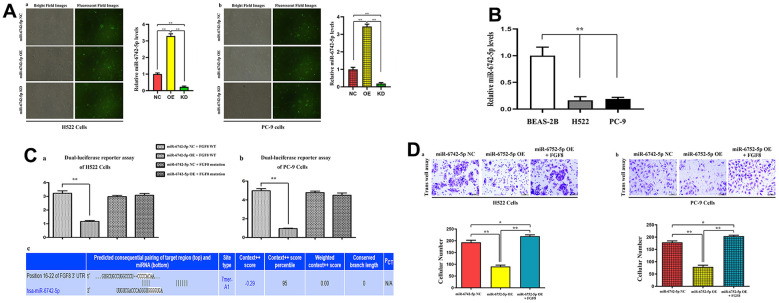
**The expression levels of miR-6742-5p and its binding for FGF8, and their interaction in tumor migratory effects.** (**A**) Quantitative real-time PCR were used to evaluate the relative expression levels of miR-6742-5p in H522 and PC-9 cells transfacted with control/NC, mimic/OE, inhibitor/KD; (**B**) the relative expression levels of miR-6742-5p in normal lung epithelial cell lines: BEAS-2B cell and H522, PC-9 cells (**C**) luciferase reporter assay was used to identify the roles of miR-6742-5p on FGF8 transcription in H522and PC-9 cells (**a**, **b**), and the putative wild-type FGF8 3'-UTR binding sequence and the mutation sequence (**c**). (**D**) the transwell assay was used to evaluate the effects of the interactions of miR-6742-5p and FGF8 in tumor migration. (**a**) ** P=0.01 miR-6742-5p OE group vs NC group; (**b**)* P=0.0481 miR-6742-5p OE+FGF8 group vs NC group.

To explore the function of miR-6742-5p in LUAD, the expression of miR-6742-5p was overexpressed or down-regulated in H522 and PC-9 cells and verified by qRT-PCR. The results of qRT-PCR displayed that miR-6742-5p was observably elevated in miR-6742-5p mimic/OE group and reduced in the miR-6742-5p inhibitor group (Figure 4Aa, b). The relative expression levels of miR-6742-5p were significantly suppressed in H522, PC-9 cells when compared with normal lung epithelial cell lines: BEAS-2B cells (Figure 4B). Additionally, dual-luciferase reporter assay verified the roles of miR-6742-5p on FGF8 transcription (Figure 4Ca, b) in H522 and PC-9 cells, and the putative FGF8 3'-UTR-WT/MUT binding sequences (Figure 4Cc). Furthermore, Transwell assays were adopted to investigate and evaluate the effects of interaction between FGF8 and miR-6742-5p OE on the migration of LUAD cells. According to the results, the number of migrated cells in miR-6742-5p mimic/OE group was smaller than that in miR-6742-5p NC group, and this effect could be reversed by FGF8 in H522 and PC-9 cells (Figure 4Da, b).

### Regulatory role of miR-6742-5p in cell invasion and migration

**Figure 5:**
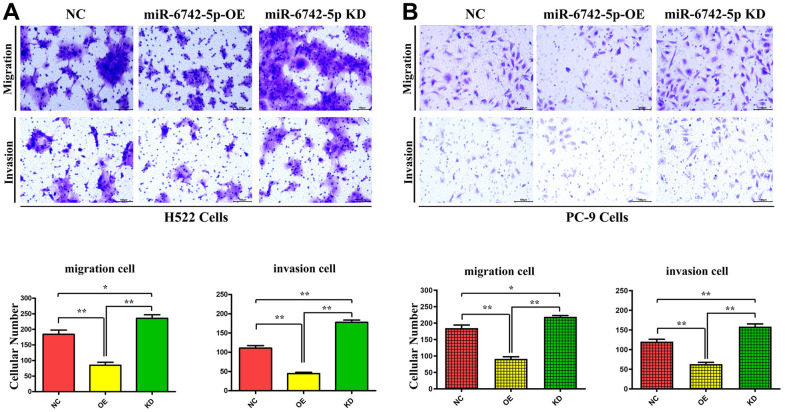
**miR-6742-5p regulated LUAD cell migration and invasion.** (**A**) H522 Cells; (**B**) PC-9 cells migration and invasion was assessed by transwell assay and its quantification. (**A**) migration **P=0.0011 miR-6742-5p OE group vs NC group;* P=0.029 miR-6742-5p KD group vs NC group; invasion** P=0.0001 miR-6742-5p OE group vs NC group;*P=0.0003 miR-6742-5p KD group vs NC group (**B**) migration **P=0.0002 miR-6742-5p OE group vs NC group;*P=0.0137 miR-6742-5p KD group vs NC group; invasion **P=0.0003 miR-6742-5p OE group vs NC group;**P=0.0063 miR-6742-5p KD group vs NC group.

**Figure 6:**
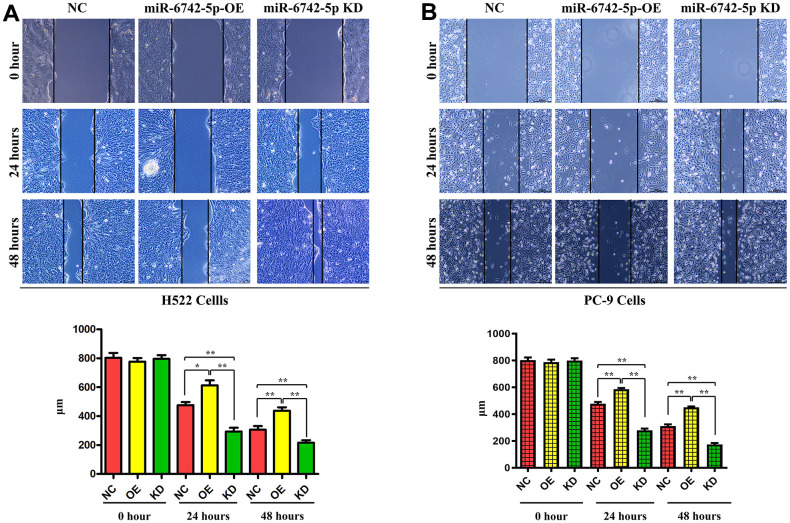
**miR-6742-5p inhibited the migratory capability of LUAD cell.** Wound healing assay showed that miR-6742 mimic (OE) significantly increased and miR-6742 inhibitor (KD) reduced wound closure and its quantifications in H522 Cells; (**A**) 24hours *P=0.0152miR-6742-5p OE group vs NC group;**P=0.0015miR-6742-5p KD group vs NC group; 48hours **P=0.0092 miR-6742-5p OE group vs NC group;*P=0.0281 miR-6742-5p KD group vs NC group (**B**) 24hours **P=0.0006miR-6742-5p OE group vs NC group;**P=0.0001miR-6742-5p KD group vs NC group; 48hours **P=0.0001 miR-6742-5p OE group vs NC group;**P=0.0002 miR-6742-5p KD group vs NC group.

To further determine the effect of miR-6742-5p on the regulation of cell migration and invasion, Transwell and wound healing assays were carried out to detect the effect of miR-6742-5p on the migration and invasion of H522 and PC-9 cells. Transwell assays proved that cell migration and invasion capacities were markedly inhibited in miR-6742-5p mimic/OE group but enhanced in miR-6742-5p inhibitor group (Figure 5A, 5B). Moreover, the wound healing assay uncovered that the cell migration ability in miR-6742-5p mimic/OE group was significantly repressed, while it was enhanced in miR-6742-5p inhibitor group (miR-6742-5p KD) ((Figure 6A, 6B). All the above-mentioned results confirmed that miR-6742-5p influenced migration and invasion of H522 and PC-9 LUAD cells.

### Regulatory role of miR-6742-5p in the invasion and migration of LUAD cells via targeting FGF8, ERK1/2, MMP9 and MMP2

**Figure 7:**
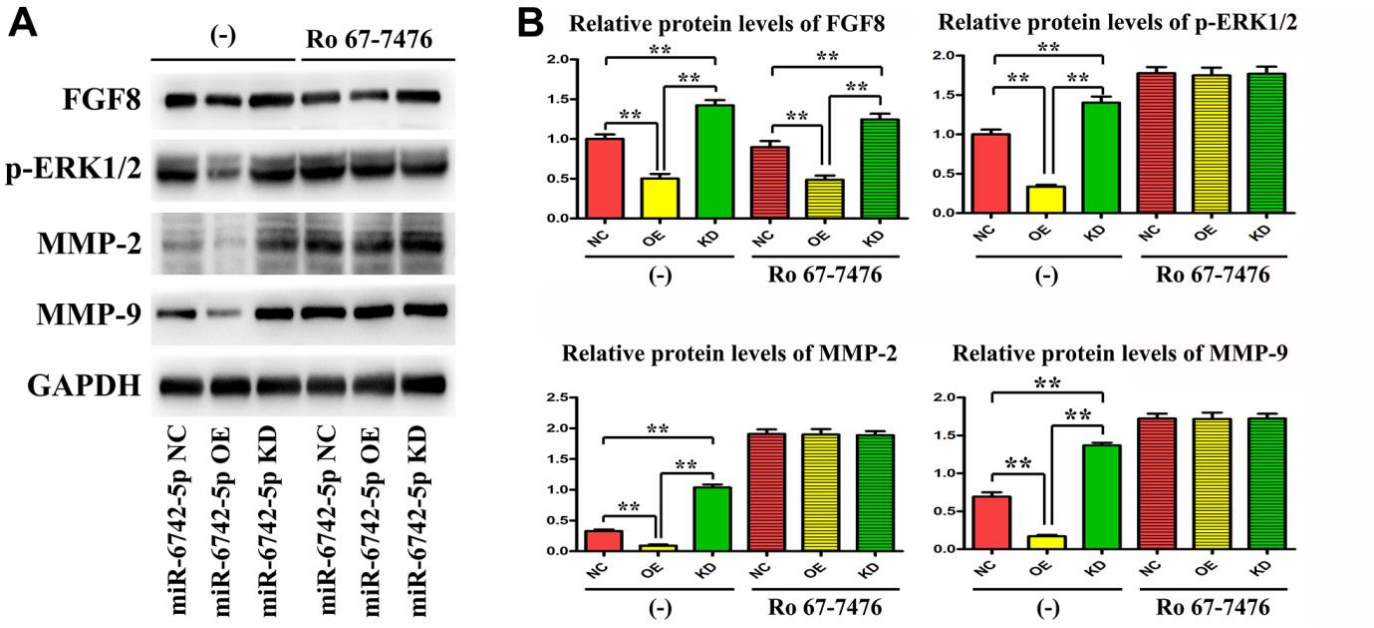
**miR-6742-5p was involved in regulating the invasion and migration of LUAD via targeting FGF8/ERK1/2/MMP9/MMP2.** (**A**) The expression of FGF8, ERK1/2, MMP9 and MMP2 was evaluated by western blot in cells treated with and without RO 67-7476 cells. (**B**) The expression of FGF8, ERK1/2, MMP9 and MMP2 quantification in cells treated with and without RO 67-7476 cells. ** P < 0.01. Relative protein levels of FGF8 (-)**P=0.0009miR-6742-5p OE group vs NC group;**P=0.0028miR-6742-5p KD group vs NC group; Ro 67-7476 **P=0.004miR-6742-5p OE group vs NC group;**P=0.0065miR-6742-5p KD group vs NC group; Relative protein levels of ERK1/2 *P=0.0134miR-6742-5p OE group vs NC group;**P=0.0063miR-6742-5p KD group vs NC group; Relative protein levels of MMP2 **P=0.0066miR-6742-5p OE group vs NC group;**P=0.0017miR-6742-5p KD group vs NC group; Relative protein levels of MMP9 **P=0.0058miR-6742-5p OE group vs NC group;**P=0.0051miR-6742-5p KD group vs NC group.

As displayed in Figure 7, the expressions of FGF8, p-ERK1/2, MMP9 and MMP2 were significantly suppressed in miR-6742-5p mimic/OE group compared with that in miR-6742-5p NC group, while they were notably promoted in miR-6742-5p inhibitor group.

Ro 67-7476 is a potent p-ERK1/2 agonist that activates ERK1/2 phosphorylation in the presence of exogenous glutamate. In the present study, Ro 67-7476 evidently attenuated the inhibitory effect on the expressions of p-ERK1/2, MMP9 and MMP2 by OE of miR-6742-5p, but had no effect on the expression of FGF8 (Figure 7), and the original data of western blot for FGF8 were shown in Supplementary Figure 6. Taken together, the above data illustrated that miR-6742-5p was involved in regulating the invasion and migration of LUAD cells by targeting FGF8, ERK1/2, MMP9 and MMP2.

## DISCUSSION

LUAD is a clinically common subtype of non-small cell lung cancer with an increasing incidence and mortality rates in recent years [1]. Despite progress in the treatment of LUAD, the prognosis of LUAD patients remains poor. Thence, further research on the pathogenesis of the progression of LUAD is of great significance for determining diagnostic biomarkers and novel effective therapeutic targets of LUAD. In this study, FGF8 was a potential target gene of miR-6742-5p and involved in the regulation of the ERK1/2 pathway. Functionally, it was discovered that miR-6742-5p regulated the invasion and migration of LUAD cells. Mechanically, miR-6742-5p regulated the invasion and migration of LUAD cells via targeting FGF8, ERK1/2 and MMP9. Thus, this study provides new insights for deeply revealing the pathogenesis of LUAD.

With the development of bioinformatics and the study of miRNAs at the molecular level of cancer biology, it can be observed that miRNAs are abnormally expressed in many human cancers [24, 25]. In addition, it has been evidenced that miRNAs play a critical regulatory role in the occurrence and progression of cancers via mediating a variety of cellular processes, including cell proliferation, apoptosis, migration and invasion [26, 27]. In fact, multiple miRNAs have been reported to be involved in the occurrence and metastasis of LUAD, and can be used as diagnostic and prognostic biomarkers of LUAD. It has been preciously demonstrated that multiple miRNAs such as miR-196b, miR-24-3p and miR-147b, are involved in the regulation of lung cancer [28–30]. In the current study, the results revealed that the expression of miR-6742-5p was decreased in human LUAD tissues and cell line compared with that in paracancerous tissues and normal lung epithelial cells (Figure 3), indicating its role in tumorigenesis. Subsequent functional analysis showed that miR-6742-5p regulated LUAD cell migration and invasion. Therefore, in-depth study on the mechanism of miR-6742-5p in LUAD is crucial for understanding the pathogenesis of lung cancer.

As presented in miRDIP and starBase databases, there were target binding sites between miR-6742-5p and FGF8 (Figure 4). Subsequently, mechanistic studies manifested that miR-6742-5p regulated the migration and invasion of LUAD cells by regulating FGF8. FGF8 is a member of the growth factor family that regulates cell growth, migration and differentiation via FGF tyrosine kinase receptor signaling [31, 32]. FGF8 expression is closely associated with the degree of degeneration in different cancer patients [33–35]. As reported, FGF8 is highly expressed in esophagogastric junction adenocarcinoma, and is expected to be a candidate gene for the prognostic factor for this cancer [36]. Besides, FGF8 also shows a high expression in oral squamous cell carcinoma tissues, and it regulates the epithelial-mesenchymal transition and induces an invasive phenotype in oral squamous cell carcinoma cells [37]. Notably, the results of this study also uncovered that miR-6742-5p influenced migration and invasion in LUAD cells by regulating the FGF8 expression, indicating that FGF8 may be a key target for regulating the LUAD progression, and the exogenous FGF8 corrected the roles of miR-6742-5p (Figure 3C). The above results identified our speculation.

As demonstrated by KEGG pathway enrichment analysis, FGF8 was likely play a role in the ERK1/2 pathway. Usually, the ERK1/2 pathway is activated in LUAD and involved in the progression of LUAD. For instance, NRG1 may be an important regulator in the development of LUAD, and its function has been found to show relevance to the ERK1/2 pathway [38]. FLOT1 promotes tumor progression, induces the epithelial-mesenchymal transition and regulates cell cycle in LUAD by regulating the ERK pathway [39]. FAM83A promotes the expression of PD-L1 via the ERK1/2 pathway, thus leading to immune escape of LUAD tumors [40]. FGF8 exerts its function mainly by binding to FGFR1 [17–19], and the activated downstream FGFR1 can directly phosphorylate the ERK1/2 [41, 42]. Moreover, the role of MMPs under the control of ERK1/2in the tumor migration or invasion and metastasis was identified. The results displayed that the MMP2/9 could rectify the inhibitory effects of miR-6742-5p KD, confirming the role of MMPs in regulating miR-6742-5p in LUAD (Supplementary Figure 4). At length, miR-6742-5p was involved in regulating the invasion and migration of LUAD by targeting the ERK1/2 pathway via FGF8.

In conclusion, miR-6742-5p is downregulated and FGF8 is elevated in LUAD tissues. The OE of miR-6742-5p inhibits LUAD cell invasion and migration and blocks the FGF8/ERK1/2 pathway. Additionally, miR-6742-5p regulates the invasion and migration of LUAD cells via targeting FGF8, ERK12, MMP9 and MMP2. This is the first study to clarify the function and mechanism of miR-6742-5p in LUAD, which may provide a new idea for revealing the pathogenesis of LUAD.

## MATERIALS AND METHODS

### Data pre-processing and differential expression analysis

A total of three datasets, namely, 2 mRNA datasets (GSE140797 and GSE116959) and 1 miRNA dataset (GSE94536), were downloaded from Gene Expression Omnibus (GEO) database (https://www.ncbi.nlm.nih.gov/). The edgeR package was utilized to convert the original microarray data into expression profile data. The differentially expressed genes (DEGs) and differentially expression miRNAs (DEMs) between LUAD and normal tissues were filtered and analyzed by the limma package in R. Later, the DEGs were subjected to hierarchical clustering analyses using pheatmap package in R (https://cran.r-project.org/package=pheatmap). Furthermore, DEMs common to the three datasets was identified using TargetScan (http://www.targetscan.org/vert_71/) and mirDIP (http://ophid.utoronto.ca/mirDIP/), so as to predict potential miRNAs that interacted with FGF8. Finally, gene set enrichment analysis (GSEA) was used to identify the pathways enriched in LUAD patients.

### Cell culture and transfection

A normal lung epithelial cell line (BEAS-2B) and LUAD cell lines (H522 and PC-9) were obtained from American Type Culture Collection (ATCC, Manassas, CA, USA) and cultured in RPMI 1640 supplemented with 10% fetal bovine serum and 1% penicillin-streptomycin (Gibco) in an incubator with 5% CO_2_ at 37° C. In addition, the miR-6742-5p negative control (NC), mimic and inhibitor synthesized by GenePharma (Shanghai, China) were transfected into the above cells lines using Lipofectamine 3000 (Invitrogen, CA, USA) based on the instructions, so as to achieve OE or knockdown (KD) of miR-6742-5p.

### Establishment of a nude mouse model of LUAD via subcutaneous injection

All the nude mice (Skbex Biotechnology, Henan, China) were adaptively fed in our laboratory for 1 week, and then randomly divided into miR-6742-5p NC group (transfected with NC) and miR-6742-5p mimic/OE group (transfected with mimic/OE, n=6). The mice in the two groups were subcutaneously injected with 100 μL of cell suspension containing 1×10^7^ H522 cells per mL in sterile PBS. One month later, all mice were euthanized, after which the subcutaneous tumors were collected, and the tumor volumes were calculated.

### Quantitative real-time polymerase chain reaction (qRT-PCR)

Total RNAs were extracted from tissue samples and cells using TRIzol kit (Solarbio, Beijing, China) following the manufacturer's protocol. Then the RNAs were subjected to reverse transcription through Reverse Transcription Kit (Thermo Fisher Scientific, Waltham, MA, USA). Next, qRT-PCR was performed using SYBR Select Master Mix (Thermo Fisher Scientific). At last, 2^-ΔΔCt^ method was adopted for the quantification of relative gene expression.

### Western blotting assay

Total proteins were isolated from tissues and cells with RIPA lysis buffer (Beyotime, Shanghai, China), and the protein concentration was conducted by a BCA kit (Beyotime). Then, the proteins were separated by SDS-PAGE and transferred onto PVDF membranes (Millipore, Billerica, MA, USA). After blocking with 5% nonfat milk for 1 h at room temperature, the membranes were incubated with primary antibodies against FGF8, ERK1/2, phosphorylated (p)-ERK1/2 and MMP9 (Cell Signaling Technology, Danvers, MA, USA) at 4° C overnight, followed by incubation with secondary antibodies at 37° C for 1 h. Finally, protein bands were visualized using ECL reagents (Beyotime).

### Transwell assays

H522 and PC-9 cells were washed and resuspended in serum-free Dulbecco's Modified Eagle Medium (DMEM) at a density of 1×10^5^ cells/mL. In the invasion assay, the cell suspension was added to the upper compartment of a 24-well Transwell plate with a Matrigel-precoated membrane (Corning, NY, USA). Meanwhile, DMEM containing 10% FBS was added into the lower compartment of the Transwell chamber. After incubation for 72 h, the invaded cells on the bottom of the lower Transwell chamber were fixed with 4% paraformaldehyde for 30 min, stained with 0.05% crystal violet for 10 min, and observed under an optical microscope (Olympus, Tokyo, Japan). In the migration assay, the cell suspension was added to the upper compartment of a 24-well Transwell plate with an 8-μm pore size membrane filter (BD Biosciences, Franklin Lakes, NJ, USA). All other processes were the same as those in the Transwell invasion assay.

### Wound healing assay

H522 and PC-9 cells were transferred to 6-well plate and co-transfected with miR-6742-5p mimic and inhibitor. When the cells reached about 90% confluence, a wound was made by scratching using a pipette tip (10 μL). Later, the migrating cells were photographed under a microscope at 0, 24 and 48 h after scratching.

### Dual-luciferase reporter assay

The potential miR-6742-5p binding site in the 3' untranslated region (3'-UTR) of the FGF8 gene was predicted using the TargetScan online database (http://www.targetscan.org). The wild-type (WT) fragment (CCCCACA) and mutant (MUT) fragment (CGGTATA) of FGF8 gene were inserted into FGF8 plasmids pmirGLO-FGF8-3'-UTR-WT and pmirGLO-FGF8-3'UTR-MUT (GenePharma). H522 cells in each group were co-transfected with the constructed WT and MUT plasmids and miR-6742-5p NC/mimic, and then they were collected after incubation for 48 h. After the addition of firefly luciferase and Renilla luciferase, the fluorescence values in each group were detected on the computer, and the ratio of Firefly luciferase to Renilla luciferase activity was calculated. Three replicates were set in each group.

### Statistical analysis

All the data were expressed as mean ± standard deviation (SD), and analyzed using ANOVA or *t*-test. P<0.05 represented that the difference was statistically significant.

## Supplementary Material

Supplementary Figures
