# Supplementary material for: miR-6742-5p regulates the invasion and migration of lung adenocarcinoma cells via mediating FGF8/ERK12/MMP9/MMP2 signaling pathway
Source: Aging (Albany NY). 2023 Jan 10;15(1):53–69. doi: 10.18632/aging.204277 (PMC9876640; doi:10.18632/aging.204277)
Supplement: Supplementary Figures [file aging-15-204277-s001.pdf]

SUPPLEMENTARY FIGURES

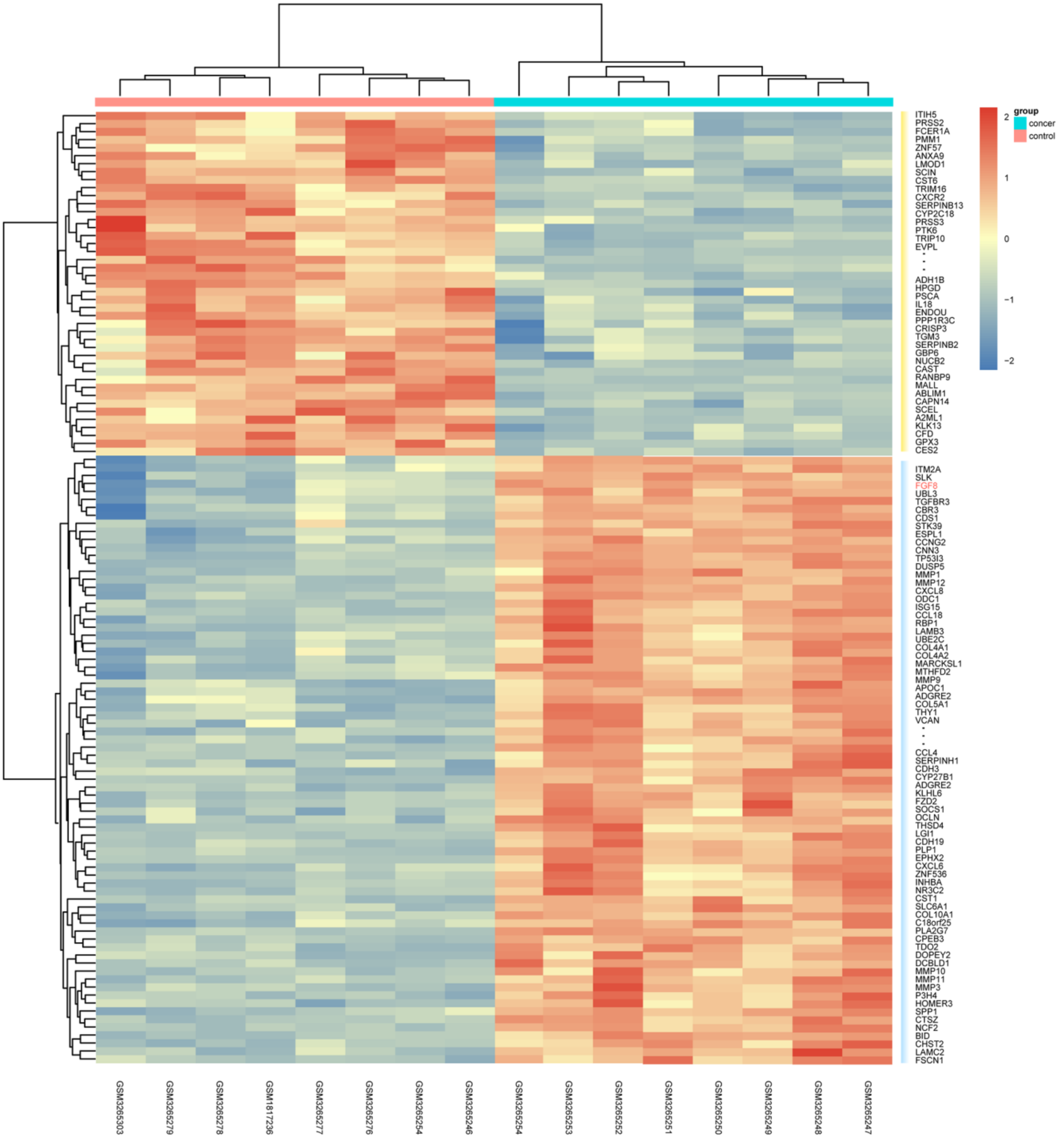

Supplementary Figure 1. The clustering analysis of differentially expressed genes between LUAD patients and normal control in GSE116959 dataset. The different colors in top column represented different groups. The green showed the LUAD and the red shows the normal control group. Red color showed overexpression and blue color represents low expression of differentially expressed genes.

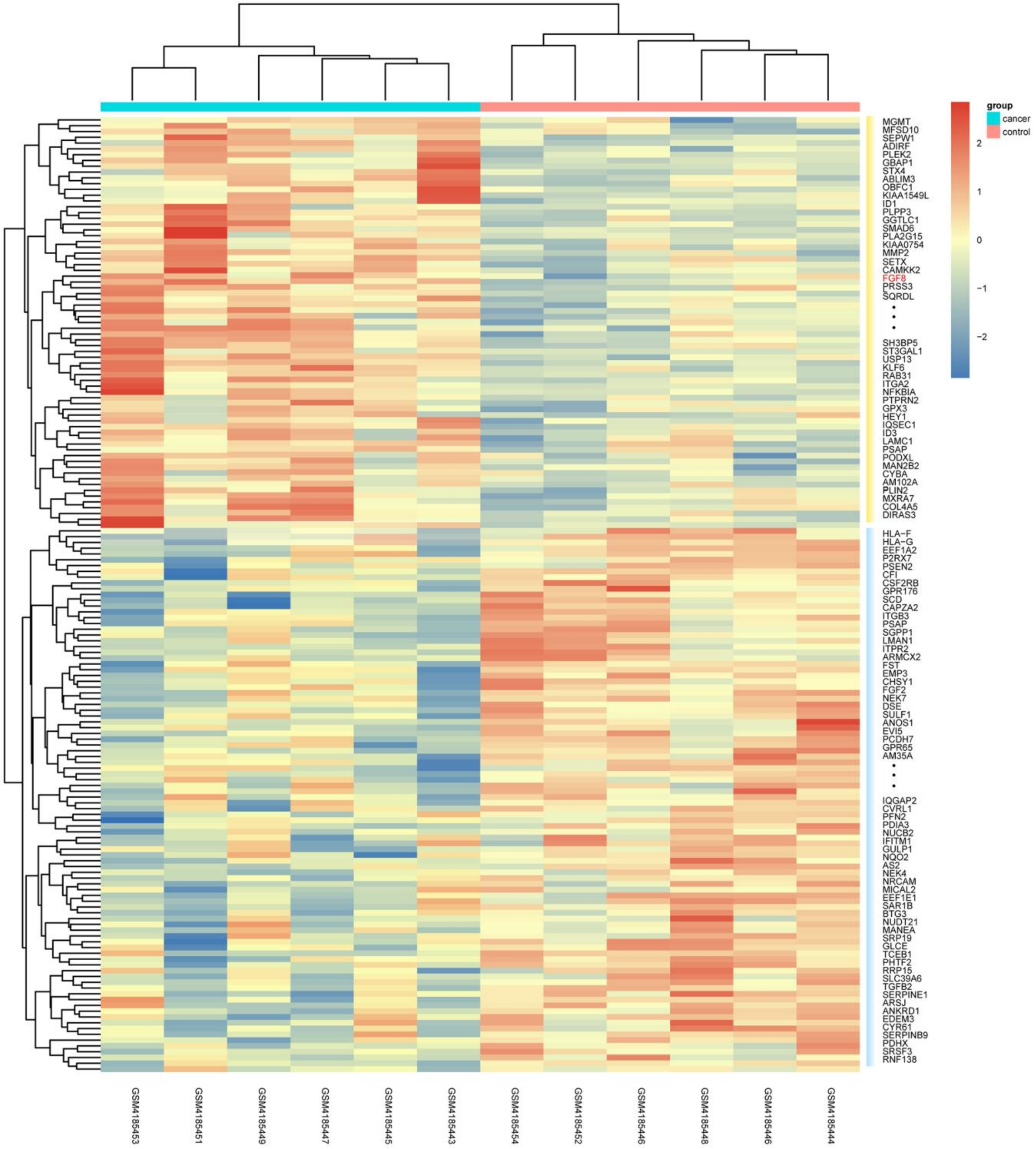

Supplementary Figure 2. The clustering analysis of differentially expressed genes between LUAD patients and normal control in GSE140797 dataset. The different colors in top column represented different groups. The green showed the LUAD and the red shows the normal control group. Red color showed overexpression and blue color represents low expression of differentially expressed miRNAs.

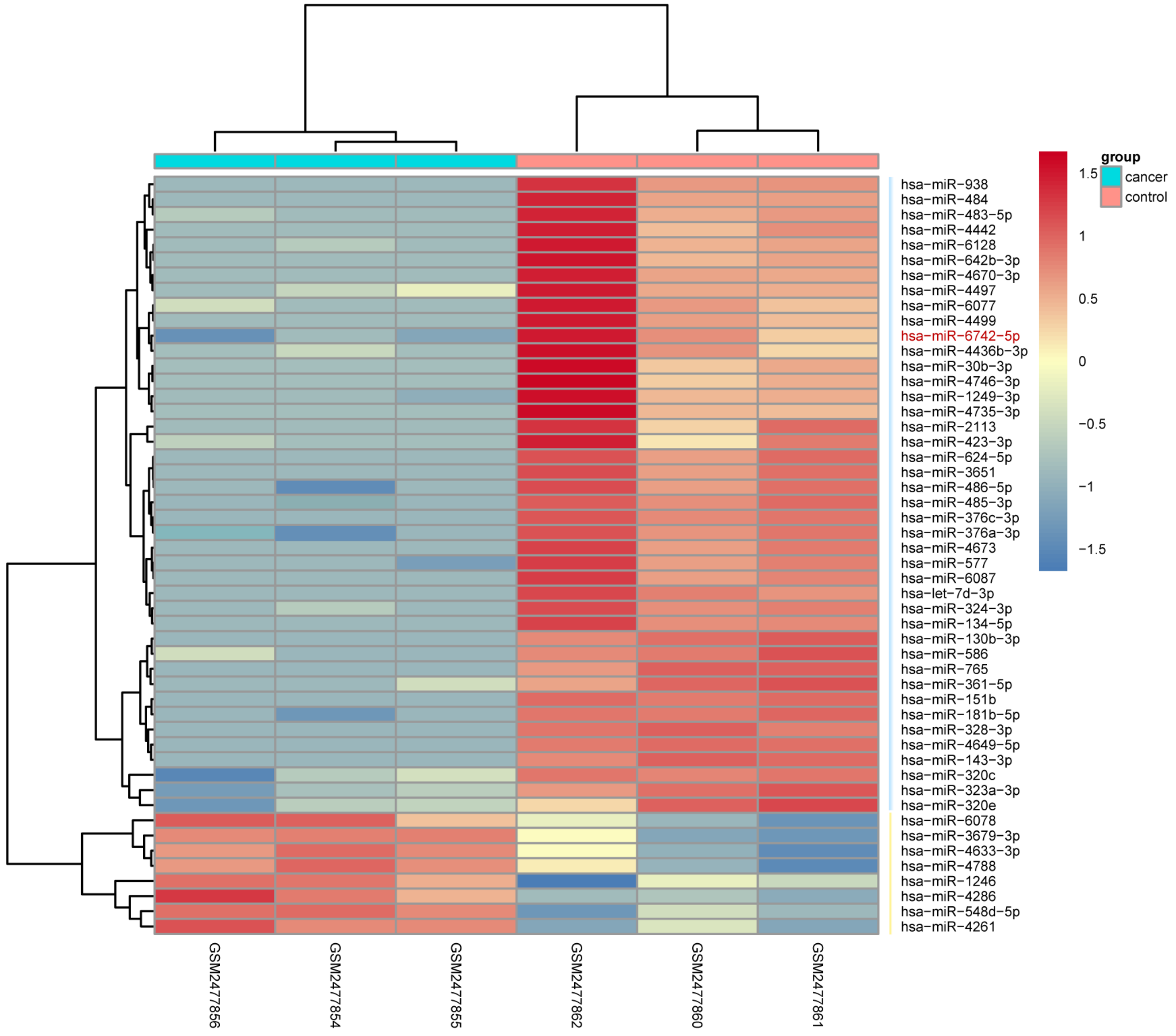

Supplementary Figure 3. The clustering analysis of differentially expressed miRNAs between LUAD patients and normal control. The different colors in top column represented different groups. The green showed the LUAD and the red shows the normal control group. Red color showed overexpression and blue color represents low expression of differentially expressed miRNAs.

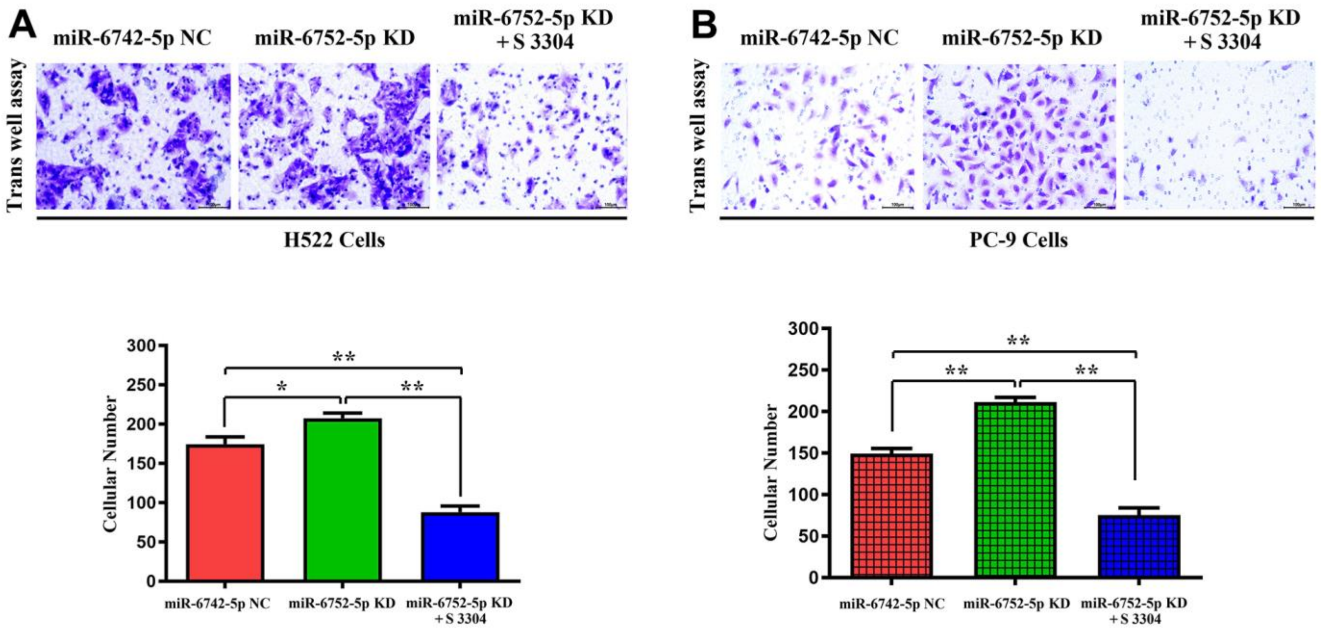

Supplementary Figure 4. The MMPs inhibitor S 3304 inhibited the miR-6752-5p KD' increased migratory abilities in LUAD. Transwell assay showed the increased migratory abilities after transfected with miR-6752-5p inhibitor, and S 3304 corrected these results (**A**) H522 Cells; (**B**) PC-9 cells and its quantification. * P < 0.05, ** P < 0.01 (magnificantion:4×). (**A**) *P=0.0281miR-6742-5p KD group vs NC group;**P=0.0004miR-6742-5p KD+S3304 group vs NC group; (**B**) **P=0.0004miR-6742-5p KD group vs NC group;**P=0.0005miR-6742-5p KD+S3304 group vs NC group.

Table: Gene sets enriched in phenotype *AN*

	GS follow link to MSigDB	SIZE	ES	NES	NOM p-val	FDR q-val	FWER p-val	RANK AT MAX	LEADING EDGE
1	BIOCARTA_ERK_PATHWAY	42	0.62	1.24	0.029	0.486	0.299	2286	tags=39%, list=18%, signal=47%
2	BIOCARTA_VEGF_PATHWAY	27	0.51	1.24	0.05	0.555	0.508	4589	tags=56%, list=37%, signal=87%
3	BIOCARTA_TGFB_PATHWAY	19	0.52	1.36	0.022	0.858	0.719	3293	tags=47%, list=26%, signal=64%
4	BIOCARTA_ALK_PATHWAY	36	0.45	1.34	0.072	0.775	0.82	1759	tags=28%, list=14%, signal=32%
5	BIOCARTA_DEATH_PATHWAY	29	0.5	1.34	0	0.663	0.843	1867	tags=31%, list=15%, signal=36%
6	BIOCARTA_CERAMIDE_PATHWAY	21	0.61	1.33	0.024	0.598	0.871	712	tags=19%, list=6%, signal=20%
7	BIOCARTA_MITOCHONDRIA_PATHWAY	19	0.55	1.33	0.036	0.518	0.871	2008	tags=47%, list=16%, signal=56%
8	BIOCARTA_RELA_PATHWAY	15	0.54	1.3	0.126	0.582	0.918	1747	tags=27%, list=14%, signal=31%
9	BIOCARTA_NO2IL12_PATHWAY	15	0.56	1.27	0.07	0.621	0.929	1643	tags=40%, list=13%, signal=46%
10	BIOCARTA_PML_PATHWAY	16	0.42	1.24	0.114	0.677	0.929	2754	tags=44%, list=22%, signal=56%
11	BIOCARTA_MCM_PATHWAY	18	0.53	1.24	0.082	0.636	0.941	1318	tags=28%, list=11%, signal=31%
12	BIOCARTA_NFKB_PATHWAY	21	0.48	1.24	0.12	0.594	0.941	527	tags=14%, list=4%, signal=15%
13	BIOCARTA_CARM_ER_PATHWAY	24	0.44	1.22	0.084	0.631	0.968	3831	tags=54%, list=31%, signal=78%
14	BIOCARTA_TID_PATHWAY	17	0.53	1.19	0.174	0.675	0.99	2230	tags=29%, list=18%, signal=36%
15	BIOCARTA_RAS_PATHWAY	22	0.53	1.19	0.242	0.669	1	2531	tags=36%, list=20%, signal=45%
16	BIOCARTA_AKT_PATHWAY	22	0.44	1.18	0.136	0.662	1	1747	tags=23%, list=14%, signal=26%
17	BIOCARTA_P53_PATHWAY	16	0.44	1.17	0.133	0.656	1	2644	tags=38%, list=21%, signal=47%
18	BIOCARTA_PITX2_PATHWAY	16	0.49	1.17	0.093	0.623	1	1725	tags=44%, list=14%, signal=51%
19	BIOCARTA_HDAC_PATHWAY	22	0.39	1.15	0.124	0.651	1	4756	tags=55%, list=38%, signal=88%
20	BIOCARTA_BARRESTIN_SRC_PATHWAY	16	0.49	1.13	0.191	0.665	1	2161	tags=31%, list=17%, signal=38%

Supplementary Figure 5. Original data for Figure 2.

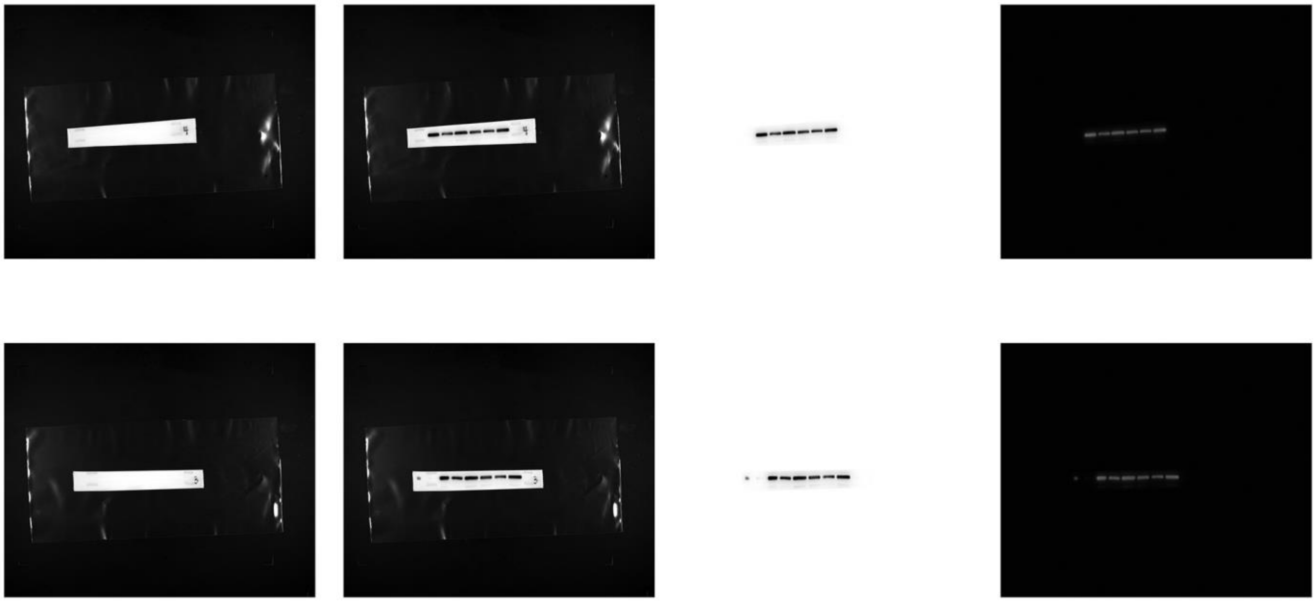

Supplementary Figure 6. Original western blot picture for FGF8.
